# Building a geographic information system (GIS) public health infrastructure for research and control of tropical diseases.

**DOI:** 10.3201/eid0104.950414

**Published:** 1995

**Authors:** A W Hightower, R E Klein


### Guidelines on the Risk for Transmission of Infectious Agents During Xenotransplants

An increasingly critical shortage of human donors has limited the availability and benefit of organ and tissue transplantation. This chronic shortage, coupled with recent scientific and biotechnological advances, has been a catalyst for new therapeutic approaches directed at using animal tissues in humans. The use of xenogeneic tissues and organs for transplantation or perfusion has raised concerns about the potential of both recognized zoonotic pathogens and unknown xenogeneic agents to infect individual human recipients and then spread through human populations.

Public health guidelines intended to minimize the risk for transmission of known pathogens through human-to-human transplantation exist. Similar guidelines addressing the issue of infectious agents that may be associated with xenotransplantation are being jointly developed by Public Health Service working groups at the Centers for Disease Control and Prevention (CDC), the Food and Drug Administration, and the National Institutes of Health. A provisional draft of these guidelines will be published in the Federal Register in late 1995. Public comment on the proposed guidelines is invited. Critical review by members of the transplant community is particularly sought. Publication of a final version of these guidelines in CDC's *Morbidity and Mortality Weekly Report* is planned for the spring of 1996.

**Louisa E. Chapman**
National Center for Infectious Diseases
Centers for Disease Control and Prevention
Atlanta, Georgia, USA

### Emerging Infectious Diseases Featured at ICAAC/IDSA Meeting

Emerging infectious diseases were highlighted recently at a joint meeting of the Interscience Conference on Antimicrobial Agents and Chemotherapy (ICAAC) and the Infectious Diseases Society of America (IDSA) in San Francisco. In his opening address, IDSA President Vincent Andriole stated that the topic of most pressing concern to both organizations was new and reemerging pathogens. Presentations were made on the following subjects: arenavirus hemorrhagic fevers; changing virulence of streptococcal infections; cryptosporidia, cyclospora, and microsporidia; dengue/dengue hemorrhagic fever; emerging fungal pathogens; epidemic diphtheria in the newly independent states; *Escherichia coli* O157:H7; *Helicobacter pylori*; human ehrlichioses; new lymphotropic herpesviruses; and rabies.

Common themes emanated from the presentations. The speakers noted that infectious diseases continue to occur throughout the world, both sporadically and as outbreaks, because of multiple factors. They observed that the incidence and prevalence of infectious diseases are increasing in certain populations, particularly among immunocompromised persons. Additionally, new infectious diseases and etiologic agents continue to be identified with remarkable frequency, and microorganisms are being identified as causes of chronic diseases, including cancer. Several presenters expressed concern about the migrations of animal reservoirs and arthropod vectors into new populations and geographic areas. The speakers also called for additional support for the public health infrastructure and for basic sciences that provide the foundation for infectious disease prevention and treatment.

### Building a Geographic Information System (GIS) Public Health Infrastructure for Research and Control of Tropical Diseases

A course on using Atlas GIS software and associated peripherals, such as digitizing tablets and global positioning systems (GPS), to build a GIS public health infrastructure in Latin American countries was taught August 7 to 18, 1995, at the Centers for Disease Control field station in Guatemala City, which includes the Medical Entomology Research Training Unit housed at the Universidad del Valle de Guatemala. The course was funded by the Special Programme on Research and Training in Tropical Diseases and presented by the Latin American Tropical Disease Research Training Consortium. Course instructors included statisticians and epidemiologists from the Division of Parasitic Diseases, National Center for Infectious Diseases, Centers for Disease Control and Prevention in Atlanta, Georgia, and in Guatemala City, Guatemala, and staff from the National Aeronautics and Space Administration, Center for Health Application of Aerospace Related Technologies, Ames Research Center, Sunnyvale, California.

Objectives of the training included the following: mastery of the principles and general concepts of all GIS systems; use of Atlas GIS/DOS to associate map files with databases to produce thematic maps, manipulate various layers (rivers, highways, village locations) of the map files to produce customized maps, create buffers around geographic features, and use them in simple analyses; designing georeferenced data files that can be read by the GIS; digitizing paper maps to acquire new data for building a GIS; use of GPS to obtain latitudes, longitudes, and elevations of villages and other major landmarks and to use this information in the GIS; and mastery of importing/exporting databases and map files.

The course was designed to enable participants to set up and use a GIS for research, planning, or operational purposes. Participating were institutions from Mexico (two teams), Colombia (two), Puerto Rico, Costa Rica, Venezuela, Guatemala (two), Ecuador, and Brazil. Each team came to the course with ideas, maps, and data pertaining to an existing project that would be continued at their home institution. Student project areas included onchocerciasis, malaria, water sanitation, leishmaniasis, and public health and natural resource utilization/preservation. The students were taught digitizing and were asked to use Guinea worm surveillance data to create their own GIS.

A full day was devoted to geographic analyses. Topics covered included aggregating data from one geographic layer to another, combining geographic features with common database values, and combining selected features to form new map layers. A workshop on remote sensing, GIS, and image classification explained that satellite imagery and remotely sensed data are obtained by measuring reflectance on seven spectral frequencies and that ground cover can be partially deduced by the amount of reflectance at each band. Field exercises to practice GPS use in the Lake Atitlan area followed. Another workshop covered advanced digitizing and gave each team a good start on the digitizing part of their projects. Individual instructions were given on how to import map files from other GIS programs into Atlas GIS. Lastly, the Guatemalan onchocerciasis GIS system was presented as a case study.

In addition to the 2 weeks of training, each participating institution received a copy of all lecture notes, the critical hardware needed to continue the project at home, and the following software, complete with documentation: Atlas GIS/DOS, Import-Export, and Arcview 2. An ongoing Internet-based discussion group for class organizers and participants is providing a forum for dialogue and monitoring of participants' progress.

**Allen W. Hightower**
**Robert E. Klein**
National Center for Infectious Diseases
Centers for Disease Control and Prevention
Atlanta, Georgia, USA, and Guatemala City, Guatemala

### APHA Session Features Emerging Infections

Emerging and reemerging infections will be the featured topic of a two-part session at the annual meeting of the American Public Health Association, October 29-November 2, in San Diego, California.

The session, titled "Emerging Infections: Solving the Mysteries in the Field and Laboratory," will focus on the worldwide impact of new and reemerging infections from both an epidemiologic and a laboratory perspective.

Eight speakers from national and international health organizations will discuss the following aspects of the public health threat of these diseases: public health strategies for controlling infectious diseases; social, geographic, ecologic, and environmental factors that have allowed these diseases to spread; the growing threat of antimicrobial resistance; the increased need for accurate and meaningful disease surveillance; and the challenge to apply the latest laboratory technology to rapidly detect and characterize new infectious agents.

**Martin S. Favero**
National Center for Infectious Diseases
Centers for Disease Control and Prevention
Atlanta, Georgia, USA

